# Methodology Considerations in Studying Mental Health, Sleep Quality, and Biopsychosocial Determinants Among Chinese and Korean Americans During the COVID-19 Pandemic

**DOI:** 10.2196/39760

**Published:** 2022-08-26

**Authors:** Jinbing Bai, Wenhui Zhang, Daesung Choi, Sangmi Kim

**Affiliations:** 1 Nell Hodgson Woodruff School of Nursing Emory University Atlanta, GA United States; 2 Rollins School of Public Health Emory University Atlanta, GA United States

**Keywords:** Asian American, gut microbiome, mental health, methodology, sleep disturbance, COVID-19

## Abstract

Asians are one of the fastest-growing racial groups in the United States. The mental health of Asian Americans, particularly regarding depression and anxiety, needs significant attention. Various biopsychosocial factors interact to influence the risks of depression, anxiety, and sleep quality among Asian Americans. Currently, multiple methodological issues exist in the research of Asian Americans, such as limited data collection using Asian languages and inconsistent reporting of race and ethnicity data, which may be lacking entirely. All these methodological issues in research may account for the seemingly low prevalence rates of mental health problems among Asian Americans. In our study on mental health and sleep quality among Chinese and Korean Americans, we adopted multiple data collection strategies during the COVID-19 pandemic, including using culturally adaptive and validated measures as well as operating culture-sensitive procedures in the recruitment and data collection. The successful use of these strategies could promote early detection and personalized treatment of depression, anxiety, and sleep disturbance among Asian Americans. These strategies would further improve health care service use in this population.

**International Registered Report Identifier (IRRID):**

RR2-10.1136/bmjopen-2020-047281

## Introduction

The racial and ethnic inequalities in mental illnesses and mental health care service use are a significant public health problem in the United States. During the COVID-19 pandemic, the prevalence of depression, suicidal thoughts or ideation, and increased or newly initiated substance use was higher among Black and Hispanic adults compared with their White peers [1]. The Healthy People 2030 goals focus on the prevention, screening, assessment, and treatment of mental disorders and behavioral conditions. One of their objectives is to increase the proportion of primary care visits where adolescents and adults are screened for depression [2].

Asian Americans are frequently underrepresented from data on mental health; thus, they remain vulnerable to inequalities in the use of mental health care services and often bear a high burden of mental disorders by tolerating them in silence. Consequently, the existing data examining their mental health and access to health care for prevention and treatment of mental health issues are limited or of poor quality [3]. When we searched on PubMed using search terms and Boolean operators such as “(depression OR anxiety) AND Asian Americans,” we retrieved only 781 articles published in the past 10 years compared with 3543 for African Americans and 4003 for Hispanics. This underrepresentation of Asian Americans in mental health research is concerning because Asian Americans are one of the fastest-growing racial groups in the United States, with a record of 23 million Asian Americans from more than 20 Asian countries [4]. Chinese and Koreans combined represent about one-third of Asians in the United States [4].

Albeit limited, the existing literature has documented widespread depression and anxiety in Asian Americans communities [5,6]. Depression is the most frequently diagnosed mental disorder in Asian American adults [6]. However, there is a huge gap between the statistics on mental health depending on the data source (whether collected nationally or locally). According to the 2019 National Survey on Drug Use and Health, Asians had the lowest prevalence of MDE in comparison to other racial and ethnic groups: 8.5% for non-Hispanic White groups, 6.3% for non-Hispanic Black groups, 6.8% for Hispanics, and 4.7% for Asians [7]. By contrast, a recent systematic review of studies among Asian Americans reported a much higher prevalence of depression than the national statistics, ranging from 26.9% to 35.6% [6]. Moreover, 10.2% of Asian Americans were reported to experience anxiety disorder [8]. Similarly, Koh [3] reported that approximately 33% and 36.9% of the Korean Americans living in Washington DC were at risk for depression and anxiety, respectively; the prevalence of anxiety was even higher compared with findings from previous studies [8]. Despite the inconsistent reporting of depression and anxiety rates among Asian Americans, they tend to manifest more prevalent, persistent, and ongoing depressive symptoms compared with their White peers [6,8]. Additionally, Asian Americans with depression or anxiety likely experience sleep disturbance [9]. As reported, Asian Americans are more likely to report interrupted sleep patterns compared with non-Hispanic White groups (33% vs 28%) [10]. These changes in sleep patterns or sleep disturbance [9] may coincide with symptoms of depression or anxiety, but they may also be caused or exacerbated by the cumulative stress related to the negative experiences of being members of a racial and ethnic minority group. For example, experiencing the effects of daily racial microaggressions is often associated with poorer sleep quality and shorter sleep duration the following day among Asian Americans [11].

Importantly, the proportion of individuals receiving treatment for mental disorders was lower among Asian Americans compared with other racial and ethnic groups. In 2019, it was reported that 51.7% of Asian adults with major depressive episode (MDE) received treatment for depression in the previous year, while 70.2% of non-Hispanic White, 59.6% of non-Hispanic Black, and 58.0% of Hispanic adults with MDE received treatment [7]. Due to the stigma attached to mental disorders and lack of culturally competent mental health services, Asian Americans are less likely than their peers to seek treatment for depression and anxiety [6,12].

### Risk Factors of Mental Health and Sleep Disturbance Among Asian Americans

**Figure 1:**
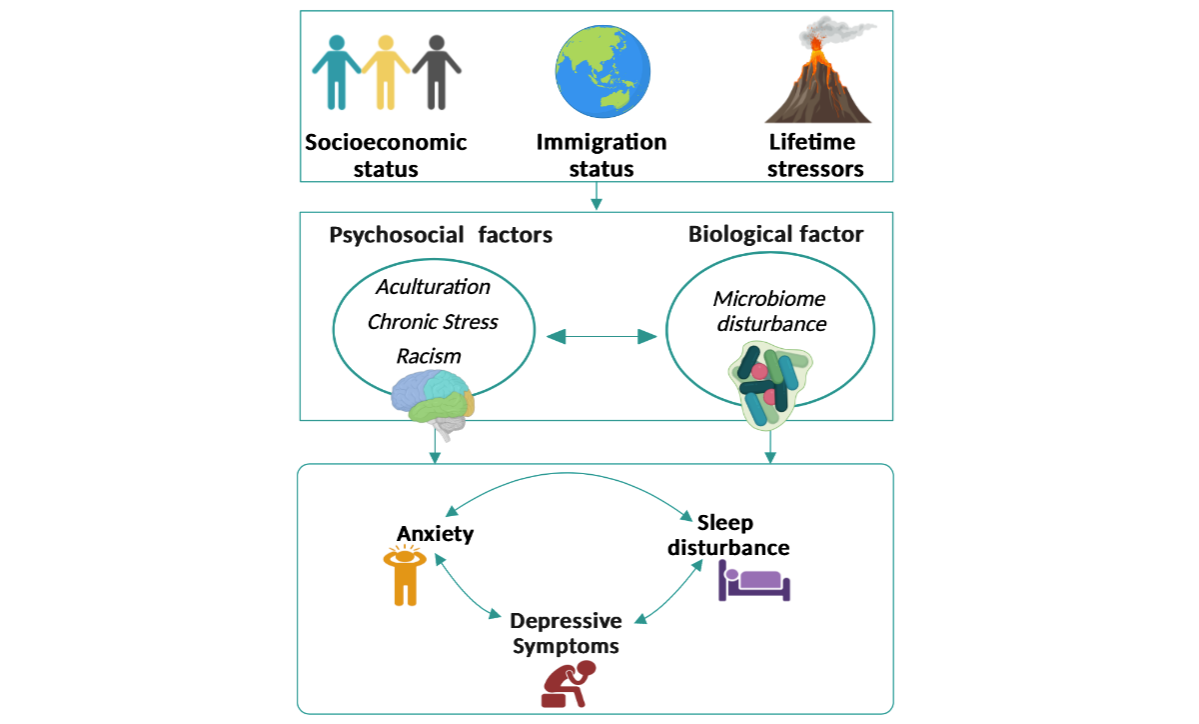
Risk factors of mental health and sleep disturbance for Asian Americans.

The risks of depression, anxiety, and sleep disturbance among Asian Americans are determined by the interactions among various psychosocial factors, including racial discrimination, lifetime stress, nativity (foreign-born vs US born), immigration status (eg, citizen, permanent resident, or undocumented immigrant), and level of acculturation [6,13,14]. Biological factors also contribute to the risk of mental disorders. To date, various biological markers have been studied to predict mental disorders (eg, anxiety and depression), such as inflammatory markers, oxidative stress, energy balance hormones, genetic, and epigenetic factors [15]. Emerging evidence suggests that the gut microbiome (ie, the gut microbes and their genomes in the gastrointestinal tract) plays a critical role in human mental health and sleep disturbance via the microbiome-gut-brain axis [16,17]. The gut microbiome is heavily influenced by an individual’s changes in lifestyle (eg, diet), stress, and geographic environment, which represent significant risk factors for depression, anxiety, and sleep disturbance during the immigration process [18]. The gut microbiome was also found to be associated with sleep disturbance [19]. Thus, studying the changes in the gut microbiome during and after migration could provide a unique opportunity to ascertain how external stimuli (eg, immigration status and lifetime stressors), psychological factors, and biological factors contribute to mental health disorders and poor sleep quality among Asian Americans during the immigration process (Figure 1). We conducted a parent study [20] to examine the relationships of psychosocial factors and the gut microbiome with anxiety, depression, and sleep disturbance among Asian Americans during the COVID-19 pandemic.

### Current Challenges to Address Mental Health and Sleep Disturbance Among Asian Americans

The fact that Asian Americans make up the lowest proportions of reported mental disorders and mental health care use in the national statistics can be viewed through the health inequity lens [21,22]. When addressing mental health among Asian Americans, we should be mindful of existing systemic barriers to accessing mental health services (eg, unavailability of culturally competent health care) [23,24] and, importantly, collecting health data from Asian Americans.

Asian Americans are viewed as a “model minority” who are better off than other racial and ethnic groups in terms of various health outcomes, including mental disorders. This entrenched stereotype contributes to the underestimated rates of depression and anxiety in this community, which may develop or be exacerbated through migration and acculturation [5] and, in turn, depression and anxiety can negatively impact their lives. Moreover, the existing literature has documented various methodological issues in collecting and analyzing data about Asian Americans. For example, the stigma toward mental health [23], linguistic mismatches between the languages of the study and study participants [25,26], and subsequently, unfamiliarity with the research process [26] may jeopardize the integrity of the research process from participant enrollment to data collection, analysis, and reporting [27]. Other methodological issues include misrepresentation and underrepresentation in research; limited data collection using Asian languages (eg, Chinese and Korean); a lack of or inconsistent reporting of race and ethnicity data in disease registries, health plans, and hospitals [27,28]; and insufficient degrees of disaggregation by subethnic group in the data [24]. Most studies categorized all Asian subethnic groups into one group, “Asians,” and generalized one subethnic group’s experience to all others due to the small sample sizes [29]. These identified issues result in a lack of high-quality data to ascertain the mental health and care use landscape, and their risk and protective factors among Asian Americans. Moreover, it is unknown to what extent the current criteria for mental disorders are valid and reliable for Asian Americans, considering that current diagnostic and assessment practices are mainly based on European or North American populations [3].

Other nonsystemic barriers encompass unique Asian cultures and experiences in the United States and diversity within the Asian population. Specifically, many Asian cultures embrace Confucianism, which discourages the open discussion of mental illness and the use of professional services [3]; they also view the body and mind as unitary rather than separate entities. Thus, the Asian population is known to express their mental distress in somatic, not psychological, symptoms, making the diagnosis and treatment of mental disorders more difficult [3]. Furthermore, because the Asian American population comprises diverse subethnic groups, such as Chinese (24%), South Indians (21%), Filipinos (19%), Vietnamese (10%), Koreans (9%), and Japanese (7%) [4], it is challenging to generalize findings on Asian Americans in general or on a particular subethnic group compared to other subethnic groups [3], particularly considering the different distributions of chronic health conditions [29].

Although different methodologies have been recommended to guide research among Asian Americans [27,28], few guidelines exist to inform the best practices to design and conduct research on mental health and sleep disturbance in this population. Therefore, the purpose of this study was to suggest methodological considerations in recruiting Asian Americans in the community during the COVID-19 pandemic and to collect more reliable data on their mental health and sleep quality, as well as their biopsychosocial risk and protective factors. These suggestions were formulated based on our experience in carrying out a parent study [20] to examine the associations of psychosocial determinants and the gut microbiome with mental health and sleep quality among Chinese and Korean immigrants. Here, a summary of our parent study is presented. Subsequently, we will discuss some best practices to design a study, recruit participants, and collect and manage biopsychosocial data from subethnic Asian American groups.

## Methodology

### Summary of the Parent Study

**Figure 2:**
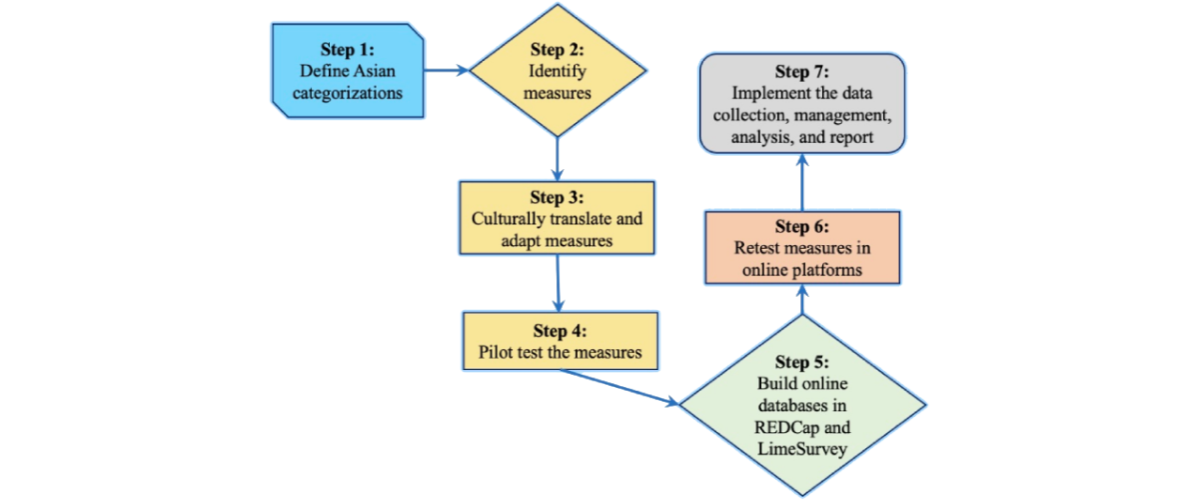
Flowchart of methodology considerations in research among Asian Americans.

This was a 1-year pilot project adopting an observational, cross-sectional study design. The study aimed to examine how psychosocial factors (eg, lifetime stress, racial discrimination, acculturation, and the gut microbiome) contribute to anxiety, depression, and sleep quality among Asian Americans [20]. Our study population was those aged 18 years or older, self-identified as Chinese or Korean, reading and speaking in English, Chinese, or Korean, and residing in Atlanta, Georgia. The participants were recruited in web-based and offline settings. The data were collected between November 2020 and May 2021, at the height of the current COVID-19 pandemic. Initially, we aimed to enroll 60 participants (n=30, 50% Chinese and n=30, 50% Korean), but we ultimately recruited 37 participants (n=17, 46% Chinese and n=20, 54% Korean). Our data collection methods included multilingual web-based surveys and biospecimen (fecal sample) collection for the gut microbiome. Specifically, we selected culturally appropriate measures with high reliability and validity for the variables of interest. We translated some of the instruments into Chinese and Korean. Subsequently, a battery of measures (Table 1) was pilot tested and delivered via web-based survey platforms, including REDCap [30] and LimeSurvey. Moreover, we shipped a stool sample collection kit to the participants’ addresses, with which they could easily collect and ship the obtained fecal samples back to the research team. All steps of this process (Figure 2) ensure a rigorous approach to studying mental health, sleep quality, and underlying biopsychosocial factors among Chinese and Korean Americans in Atlanta, Georgia, particularly during the COVID-19 pandemic.

Of importance, our study attempted to address the methodological issues mentioned earlier. First, our target populations were Chinese and Korean adults. Acknowledging potential subethnic differences in many characteristics, we analyzed both aggregated and disaggregated data to examine the associations between biopsychosocial factors and mental health and sleep quality. Second, we made recruitment materials, web-based surveys, and study instructions available to participants in 3 languages (English, Chinese, and Korean). Third, when choosing the survey instruments, we tried to select those designed to reflect Asian American experiences. Lastly, our research team comprised Chinese and Korean bilingual investigators fluent in English and Chinese or Korean. We also strategically employed a coprincipal investigator system, with one principal investigator being Chinese and the other being Korean, to equally represent both subethnic groups’ perspectives and lived experiences from the study design to the data analysis stage. The Chinese investigators communicated with Chinese-speaking participants, and the Korean investigators did the same with Korean-speaking participants. We believe these collective efforts increased the accuracy of the measured characteristics among Chinese and Korean Americans.

**Table 1:** Study measures.

Variable, measure, and instrument					Linguistic version				
					English		Chinese		Korean
**Sociodemographic and clinical factors**									
		Demographics Short Form (eg, sociodemographic characteristics, health behaviors, and medical history)			X^a^		T^b^		T
**Psychosocial factors**									
	**Acculturation**								
			Nativity (foreign-born vs US born, duration of US residence)	X		T		T	
			Suinn-Lew Self Identity Acculturation Scale	X		T		T	
	**Stress**								
			Adult STRAIN^c^	X		T		T	
			Acculturative Stress Scale	X		X		X	
			Subtle and Blatant Racism Scale for Asian Americans	X		T		T	
	**Diet**								
			PrimeScreen Survey	X		T		T	
**Biological factor**									
	**Gut microbiome**								
			Fecal specimen	X		T		T	
**Outcomes**									
	**Depression**								
			PROMIS^d^ Short Form–Depression	X		X		X	
	**Anxiety**								
			PROMIS Short Form–Anxiety	X		X		X	
	**Sleep disturbance**								
			Pittsburgh Sleep Quality Index	X		X		X	

^a^X: available versions of the measures.

^b^T: translated versions if needed.

^c^STRAIN: Stress and Adversity Inventory.

^d^PROMIS: Patient-Reported Outcomes Measurement Information System.

### Ethics Approval

All participants provided written informed consent. Ethics approval was provided by the Emory University Institutional Review Board (IRB #: STUDY00000935).

### Methodology Considerations in Studying Mental Health and Sleep Disturbance for Asian Americans

Figure 2 describes the flowchart of a 7-step procedure in studying mental health and sleep disturbance among Asian Americans. Details included how we defined a target population of Asian Americans; identified appropriate survey measures; translated, culturally adapted, and pilot tested the selected survey measures; built a web-based database for multilingual surveys; retested the survey measures on web-based platforms; and finally collected, managed, and analyzed the data.

#### Step 1: Defining a Target Asian Americans Population

Currently, most surveys lack or have limited subgroup categorizations for Asian Americans in terms of nativity, subethnicity, and geolocation [27]. In our study, we defined Asian Americans as those self-identified as Chinese or Korean, including the first- and second-generation Chinese and Korean immigrants, and residing in the Greater Atlanta area, Georgia, US. First-generation immigrants are foreign-born and living in the United States, regardless of the duration and purpose of residence in the United States. Second-generation immigrants are native-born and currently living in the United States. Recent studies using large-scale national epidemiological surveys have demonstrated that the prevalence of depressive symptoms varies in different subgroups based on immigration-related characteristics [31]. The diversity loss and composition changes in the gut microbiome are determined by changes in the geographic living environment [32], diet acculturation [32,33], risk factors (eg, chronic stress [34] and racial discrimination [35]), and protective factors (eg, stress resilience [36] and social support [37]). Thus, it is important to recruit specific subethnic Asian American groups with different immigrant generations due to their distinct identities and lived experiences that may shape their risks of mental health and sleep disturbance.

#### Step 2: Identifying Appropriate Measures

Validated measures are essential tools to ensure reliable and accurate assessment and study results [38]. After finalizing the research questions and the study population, the next step is identifying appropriate measures that promote cultural competency by taking Asian Americans’ language proficiency into account. Specifically, these are appropriate measures taken for languages available in mother tongues (eg, Chinese and Korean) of the target populations. Choosing the right instruments for studies can be challenging when various measures are available to assess depression, anxiety, and sleep quality, while all measures have their own unique pros and cons. To overcome these difficulties, we used the instrument selection and evaluation criteria (ie, reliability, validity, standardization, and practicality [39]; Table 2) and selected the 6-item Patient-Reported Outcomes Measurement Information System (PROMIS) Short Form–Depression [40], the 6-item PROMIS Short Form–Anxiety [41], and the 10-item Pittsburgh Sleep Quality Index [42] for our outcome variables (depression, anxiety, and sleep disturbance). Using the existing guideline made the instrument selection process less daunting and more systematic.

**Table 2:** Instrument assessment and evaluation criteria.

Criteria	Definition
Reliability	The degree to which “...scores for people who have not changed are the same for repeated measurements, under several situations,” including test-retest reliability, intrarater and interrater reliability, and internal consistency [43].
Validity	The degree to which an instrument truly measures the constructs it purports to measure, including content and face validity, criterion validity, and construct validity [44].
Standardization	The degree to which it can be used across persons so that the administration instructions, content format, and scoring procedures are predetermined and identical no matter who administers and undertakes the scoring.
Practicality	To assess whether the measure is lengthy to complete or complex to score.

Subsequently, we asked the original developers for approval to use those instruments. These 3 measures are available in English, Chinese, and Korean, with excellent reliability and validity among various populations, including Asian Americans [40-42]. We paid a relevant copyright fee to use the Chinese and Korean versions of PROMIS measures and secured corresponding scoring sheets for each measure. Notably, despite the availability of alternative measures, which were free of charge, we selected the PROMIS measures for depression and anxiety to produce data comparable with existing data since the PROMIS, funded by the National Institutes of Health, successfully addressed the lack of standardization in patient-reported outcomes, among which little comparability exists [45].

Similar instrument selection and evaluation criteria [20] were used to select measures for the psychosocial determinants, as with as the outcome variables (Table 1). These measures included the Suinn-Lew Self Identity Acculturation Scale (to measure acculturation), Acculturative Stress Scale (to measure acculturative stress), Subtle and Blatant Racism Scale for Asian Americans (to assess interpersonal racial discrimination), and Adult STRAIN (Stress and Adversity Inventory; to assess lifetime stress). Confounding factors such as sociodemographic, medical, and behavioral characteristics, as well as diet, were measured by the demographic short form and the PrimeScreen (a brief dietary screening tool), respectively. These instruments have been previously validated, and the English versions are widely used. Some of these measures required translation or adaptation for use in Asian Americans, as discussed below. Lastly, biological data of the gut microbiome were collected using fecal specimens. The sample collection was performed following the Human Microbiome Project protocol [46]. The adapted version of the gut microbiome data collection has been tested among adults [47] and children with chronic illnesses [48].

#### Step 3: Translating and Culturally Adapting the Selected Measures

Due to the limited use of Asian languages in data collection [27], our study provided participants with the aforementioned self-report measures in 3 languages (English, Chinese, and Korean) to decrease sampling bias from the high rates of limited English proficiency and linguistic isolation (defined as living in a household in which all members aged 14 years and older speak a non-English language and also having difficulty with English [49]) in Asian Americans [50].

For measures that had not yet been translated into Chinese or Korean, we contacted the instrument developers to obtain permission to use and translate them. Following the instrument translation and cross-cultural adaption guideline from the World Health Organization [51], the Subtle and Blatant Racism Scale for Asian Americans, Adult STRAIN, and PrimeScreen were translated into Chinese and Korean versions. The instrument translation process included forward translation, expert panel back translation, pretesting, and finalizing the measures [51].

We formed a bilingual instrument translation team, which included 3 research team members and 1 external member with extensive research expertise in sociology, Asian immigrants, stress, and mental health. All these members were fluent in English and Chinese or Korean. After one team member translated all the instruments into Chinese or Korean versions (forward translation), another member translated them back into English versions (backward translation). Subsequently, both members compared the original English and back-translated English versions to evaluate the semantic equivalence of the translation. Consensus discussions between these 2 members resolved discrepancies in the translation and meanings to ensure conceptual equivalence across the translations. This instrument translation process ensures the accuracy and validity of instruments across diverse populations [52]. Regarding collecting fecal specimens for the gut microbiome, all the instructions for participants, standard operating procedures, as well as home-based sample collection and return shipping instructions were prepared in English initially and then translated into Chinese and Korean. The Chinese and Korean versions of the documents were reviewed and tested by bilingual research team members before initiating the data collection.

#### Step 4: Pilot Testing the Measures

Multiple procedures can be used during the instrument translation process to test the final translated instrument for clarity, comprehensiveness, appropriateness, or cultural relevance, such as a monolingual test (ie, examination of the target language version among monolingual subjects) and a bilingual test (ie, examination of both source and target language versions among bilingual subjects) [52]. Current approaches to the instrument test vary. Our study tested the translated instruments among bilingual subjects speaking English and Chinese or English and Korean. Three first-generation Asian immigrants in the research team tested each language version of the same instrument to identify potential issues in administration. Lastly, our protocol for collecting fecal specimens did not need pilot testing since it has already been demonstrated to work well in our previous studies [47]. In short, we used pictorial and written step-by-step instructions to coach participants to obtain their fecal samples using the home-based stool specimen collection kit.

#### Step 5: Building a Web-Based Database for Multilingual Surveys

The COVID-19 pandemic has led to a global shift in clinical research methodologies. Many remote data collection approaches (eg, web-based survey platforms, group conference calls, and phone calls) have been widely adopted by researchers to mitigate the impact of restrictions in research activities [53]. As a result of working collaboratively with various partners at our institution such as the IT department, Office of Nursing Research, and Institutional Review Board, the consent form, data collection measures (both original and translated versions), and data management were seamlessly connected between 2 different web-based survey platforms (REDCap and LimeSurvey). This enabled all participants to provide their consent and complete the web-based surveys easily. Importantly, our bilingual team members assisted the participants in their preferred languages throughout recruitment and data collection.

After pilot testing the instruments, we built web-based surveys in 3 languages (English, Chinese, and Korean) on REDCap [30], a web-based software and workflow methodology for designing, collecting, and managing clinical and translational research databases. Then, we consulted a data manager at the Office of Nursing Research to help build the different versions of the web-based surveys in a coordinated manner, with all 3 language versions of the questionnaires being contained in one REDCap project. Our structure of the web-based surveys—multiple language versions on a single platform—caused many challenges. First, we needed to link the participant’s screening survey with the main survey in the language the participant initially selected to use. It was problematic because we had 1 screening survey shared by all participants speaking different languages and 3 language versions of the main surveys. The screening survey asked the participant to click on the preferred language: “English,” “한국어,” and “中文.” Thus, we had to create a branching logic to connect, for example, an answer saying “한국어” with the main survey in Korean. Second, because all 3 versions of the questionnaires were built one after another (English questionnaires appeared first, followed by Korean and Chinese questionnaires), we needed an algorithm for each language survey to stop before it automatically moves to the next questionnaire in a different language. Lastly, although our main survey platform was REDCap, the Adult STRAIN was built by a developer on LimeSurvey. We had to link REDCap and LimeSurvey, which was crucial since lifetime stress exposure assessed by the Adult STRAIN was one of our study’s key variables of interest. We were concerned about possible dropouts if 2 survey platforms were sent to the participants, as they might be willing to complete one survey but not the other. As a solution, we embedded the Adult STRAIN’s URL at the end of the REDCap survey so that the participants were seamlessly led to the Adult STRAIN survey. Since we had to link the data from REDCap and LimeSurvey through a unique survey ID assigned to each participant, we created a field in REDCap before the transition to LimeSurvey to inform the participant of their unique REDCap survey ID by using the piping function. The Adult STRAIN asked the participants to enter the study ID. By entering the REDCap survey ID in that field, we could link both surveys for each participant.

#### Step 6: Retesting the Measures on Web-Based Platforms

The final step was to retest our measures in the form of web-based surveys before moving them from the testing stage to production stage and to identify and resolve any issues regarding transitions of questionnaires, transitions between 2 web-based platforms (REDCap and LimeSurvey), and mismatches between the participant’s selected language and the language of the survey. The same 3 bilingual research team members independently tested the 3 language versions of the web-based surveys. This process validated the web-based surveys’ feasibility, integrity, and usability from the participant’s perspective. All the issues identified during the retesting process were addressed by making the necessary modifications to the surveys, and the process was tested repeatedly until we were confident to launch the web-based surveys.

#### Step 7: Collecting, Managing, and Analyzing Data

During the implementation stage, several strategies were suggested to improve research engagement with potential participants and reduce mistrust that Asian immigrants might have toward research. First, a multilingual research team was built to promote participant accrual and data analysis. Our team included members with a Chinese or Korean cultural background who could fluently speak English and either Chinese or Korean. In addition, our team members had extensive research experience working with the Asian populations of interest. Second, we established an advisory board comprising academics with expertise in immigrant populations and mental health, as well as community members from churches and clinics, such as a Korean pastor and a Chinese clinician (nurse practitioner). All the community members were Chinese or Korean and served Chinese or Koreans in the Greater Atlanta area. The goal of the advisory board was to improve the research team’s engagement with and accessibility to the target Asian populations. Third, our data collectors or points of contact were matched with participants by the subethnic identity and language, which enabled the team to answer the participants’ questions or address concerns in a culturally sensitive fashion.

Lastly, a detailed standard operating procedure was created to collect and manage the web-based surveys and biological data. When potentially eligible participants contacted the research team, our bilingual team members emailed them to make an appointment for a brief screening call. During the screening call, if participants met the inclusion criteria and remained interested, the researchers obtained their verbal consent to take part in the study. Due to the COVID-19 pandemic, there were no in-person interactions with participants. Upon obtaining participants’ informed consent, the bilingual team members emailed them a link to the REDCap web-based survey. The participants completed the web-based survey in their preferred language. They provided their contact information (eg, name, mailing address, phone number, and email address) in the screening survey, where we specified why this information was requested. For example, participants’ names and mailing addresses were used to ship the microbiome data collection kits, including pictorial and written instructions in English, Chinese, or Korean. Participants’ phone numbers were used to receive their verbal consent, and email addresses were used to send the study compensation upon the subjects’ completion of the study procedures. Our research staff also sent weekly reminders prompting the participants to return their stool samples to the research team.

All self-reported subject data were stored on REDCap and LimeSurvey (Adult STRAIN only). Before statistical analysis, all the data were reviewed for quality, distributions, and missing data bias. Descriptive analyses, including count, percentage, mean, and standard deviation, were used to describe the participants’ characteristics and outcome variables including anxiety, depression, and sleep disturbance. When the data were not normally distributed, we used nonparametric statistics, including the Mann-Whitney *U* and Spearman correlation tests. Spearman correlation was used to examine the associations among anxiety, depression, and sleep problems. For the biological data, the stool samples were placed in −80 °C freezers until DNA extraction. Based on the Human Microbiome Project protocol, the microbial DNA was extracted from fecal specimens using the Power Soil isolation kit (MO BIO Laboratories, Carlsbad, CA, US). The 16S rRNA V3-V4 gene regions [54,55] were extracted and sequenced by Emory University’s Integrated Genomics Core. All analyses were conducted using Quantitative Insights into Microbial Ecology 2 [56-58] and R 3.3.3 (R Foundation for Statistical Computing).

## Discussion and Conclusion

In our study, the data collection methods—multilingual web-based survey (English, Chinese, and Korean) and biospecimen collection (gut microbiome)—were well accepted by Chinese and Koreans, and no specific concerns or inconveniences were reported by the participants. Within 5 months, 37 Chinese and Korean immigrants were recruited and finished the self-reported data collection, and 21 (57%) of them provided the gut microbiome data.

While recruiting Asian Americans was somewhat challenging, it was still feasible to study their mental health and sleep quality during the COVID-19 pandemic. During the pandemic, Asian Americans have been a target of racial discrimination, including aggression (eg, verbal threat) and physical violence. Those residing in the Greater Atlanta area were especially impacted, either directly or indirectly, by the Atlanta spa shooting by an armed White male who killed 8 women of Asian descent in March 2021. These racially targeted incidents and the social justice movement across the United States might encourage Asian Americans to pay more attention to their mental health and sleep problems, thus convincing Asian Americans to be more willing to participate in this study or other similar studies. Moreover, we found it acceptable to examine both the biological (ie, gut microbiome) and psychosocial (eg, lifetime stress, racism, and acculturation) determinants through noncontact measures in the Chinese and Korean communities.

This paper discussed the current methodological issues in researching Asian Americans’ mental health and sleep quality and suggested multiple solutions, based on our parent study, to mitigate such research problems at the design stage. Islam et al [27] have summarized methodological issues for collection, analysis, and reports of large data sets in Asian Americans, such as US Census, American Community Survey, and the National Health and Nutrition Examination Survey. Poor sampling and low representation of the Asian American population were critically addressed in national-level data sets. Additionally, very limited existing data combined both biological and psychosocial determinants together to contribute to mental health outcomes among Asian Americans [20]. To address these methodological issues, we specifically proposed narrowly defining Asian Americans as a study’s target population; selecting culturally adapted and validated measures; translation and reiterative testing; using practical data collection methods (eg, web-based survey, collecting samples at home, and shipping back the samples with prepaid postage); and preparing for culturally appropriate standard operating procedures for recruitment, data collection, and study management. The proposed methods could serve as a guide to other researchers investigating the role of biopsychosocial determinants in mental health and sleep disturbance among Asian Americans.

This study has several limitations to be addressed. Our parent study focused on the mental health and sleep quality in a healthy population. Participants’ physical status, such as disease conditions, may present further methodological issues in studying Asian Americans. The 7 steps proposed in this study should be further evaluated among Asian Americans with different care needs. In addition, the best practices of methodology were derived from a pilot study (eg, small sample size and geographic area limitation) among Chinese and Korean Americans. Our study methodology and results may not be generalizable to other Asian American groups, such Vietnamese and Filipino, and this methodology should be carefully evaluated in other Asian subethnic groups. Lastly, the best practices from small-scale studies of Asian Americans should be compared with national-level data sets to confirm the validity of the methodology practices and thereby ensure the rigor of the findings.

In conclusion, this study demonstrated some best practices for design, recruitment, data collection, and analysis among Asian Americans based on an exemplar parent study conducted during the COVID-19 pandemic. Implementing best practices will provide high-quality data that enable us to determine more accurately the landscape of mental health inequities affecting Asian Americans.

## Abbreviations

MDE: major depressive episode
PROMIS: Patient-Reported Outcomes Measurement Information System
STRAIN: Stress and Adversity Inventory
